# Crypt4GH: a file format standard enabling native access to encrypted data

**DOI:** 10.1093/bioinformatics/btab087

**Published:** 2021-02-05

**Authors:** Alexander Senf, Robert Davies, Frédéric Haziza, John Marshall, Juan Troncoso-Pastoriza, Oliver Hofmann, Thomas M. Keane

**Affiliations:** European Bioinformatics Institute, Wellcome Genome Campus, Hinxton CB10 1SD, UK; Enthought, Inc., 200 W Cesar Chavez, Suite 202, Austin, TX 78701, USA; Wellcome Sanger Institute, Wellcome Genome Campus, Hinxton CB10 1SA, UK; Centre for Genomic Regulation (CRG), The Barcelona Institute of Science and Technology, Barcelona 08003, Spain; Wolfson Wohl Cancer Research Centre, Institute of Cancer Sciences, University of Glasgow, Glasgow G61 1QH, UK; School of Computer and Communication Sciences, EPFL (Ecole polytechnique fédérale de Lausanne), EPFL IC IINFCOM LDS, BC 266 (Bâtiment BC), Lausanne CH-1015, Switzerland; University of Melbourne, Centre for Cancer Research, Victorian Comprehensive Cancer Centre, 305 Grattan Street, Melbourne, VIC 3000, Australia; European Bioinformatics Institute, Wellcome Genome Campus, Hinxton CB10 1SD, UK; School of Life Sciences, University of Nottingham, Nottingham NG7 2RD, UK

## Abstract

**Motivation:**

The majority of genome analysis tools and pipelines require data to be decrypted for access. This potentially leaves sensitive genetic data exposed, either because the unencrypted data is not removed after analysis, or because the data leaves traces on the permanent storage medium.

**Results:**

: We defined a file container specification enabling direct byte-level compatible random access to encrypted genetic data stored in community standards such as SAM/BAM/CRAM/VCF/BCF. By standardizing this format, we show how it can be added as a native file format to genomic libraries, enabling direct analysis of encrypted data without the need to create a decrypted copy.

**Availability and implementation:**

The Crypt4GH specification can be found at: http://samtools.github.io/hts-specs/crypt4gh.pdf.

**Supplementary information:**

Supplementary data are available at *Bioinformatics* online.

## 1 Introduction

The number of human genomes sequenced has increased dramatically in recent years, supported by the falling cost of sequencing and the availability of portable sequencing machines. Human genetic data must be kept secure at all times. This includes storing and archiving the data, transporting the data between authorized parties, and viewing or analyzing the data. This requires increased security to protect data access and greater care handling data in permanent storage. Furthermore, all data leaves a footprint on permanent storage (even after they are deleted) so data must be disposed of in a secure manner when hardware is replaced.

There are a variety of solutions for keeping (genomic) data secure, each addressing different aspects of data handling. Cryfa (Morteza et al., 2019) is a software tool aimed at encrypting genomic data while also compressing it by taking advantage of the known structure of common genomic file formats. The goal of Cryfa is to enable secure storage/archiving of genomic data. The SECRAM framework (Zhiconget al., 2016) provides a specification for combining position-based compression and encryption. It allows for selective retrieval of information with privacy-preserving features such as using multiple encryption keys to restrict access to sections of the data file. Some solutions based on homomorphic encryption (Kim *et al.*, 2020) or secure multi-party computation are aimed at keeping data secure even during the computational phases, although these approaches are not general purpose, as they have to be specifically tailored to concrete applications. While each of these approaches is effective at securing data at a particular phase of the data’s life cycle, no single encryption method keeps data secure at all times or preserves the structure of the underlying files. Each approach requires significant effort to alter existing workflows or modify software tools, which limits adoption at a large scale.

Recently approved by the Global Alliance for Genomics and Health (GA4GH), Crypt4GH is a random-accessible file format specification that allows for reading encrypted data from file or remote APIs and performing in-memory decryption of just the byte ranges needed. This approach considerably reduces the attack surface, therefore mitigating the risks of decrypting the whole file and/or storing decrypted data in disk. By transparently enabling existing workflows to use encrypted data natively we hope to increase their scope and usage, including both research and clinical settings. Crypt4GH is not intended to address all aspects of genomic data security (e.g. key management) and should form part of an overall data security strategy.

## 2 Materials and methods

Crypt4GH acts as a container around existing sequencing files, meaning that the format of the underlying data remains unchanged to maintain compatibility with existing readers and indexes. A Crypt4GH file consists of a header and a data portion (Supplementary Fig. S1). The header is encrypted with an individual recipient’s public key and contains the secret symmetric encryption key necessary to access the data portion. A message authentication code (MAC), based on a shared secret key, is used as an integrity check and prevents decryption if the recipient cannot validate it. Using authenticated encryption in individual segments mirrors solutions like Transport Layer Security (TLS) (Rescorla, 2018) and prevents undetected modification of segments. Dividing the file into fixed-size (except at the end) blocks enables random-access into the encrypted file. The header contains unencrypted fields identifying the format with magic and version numbers, as well as a count of header packets. The header packets are encrypted with the help of a user’s public key and contain information necessary to access the data portion of the file. Multiple encryption methods can be specified, whilst only one is currently supported: ChaCha20-Poly1305. This simplifies implementations while allowing the current choice to be replaced in a future revision if it is found to have any vulnerabilities. The criteria for choosing encryption methods included having strong security guarantees, widespread library support, and being used in other common standards such as TLS version 1.3 (see security review document—Supplementary File S1). There are currently three implementations of Crypt4GH: (i) embedded within htslib and part of Samtools, supporting both reading and writing of Crypt4GH files; (ii) a Java encryption/decryption tool and extensions to htsjdk enabling read and write of Crypt4GH files; and (iii) a Python encryption/decryption tool (see Supplementary Materials).

## 3 Results

We benchmarked the performance of reading/writing both plain and Crypt4GH format BAM from Illumina sequencing data of *S.cerevisiae* (ERR065185, 1.08 Gbp) and VCF from the 1000 Genomes Project (Chr 13, 2.8M variants) (Table 1). Our tests operated in two modes: full file reading, and random access retrieval of genomic loci. For full file reading, we observed an overhead of between 21% and 30% for BAM and 0.3% and 0.9% for VCF files compared to plain files. For random access, the overhead was between 22% and 42% for BAM and 1% and 3% for VCF. These are worst-case numbers showing purely data access times; any workflows using the data will proportionally decrease the overhead of accessing Crypt4GH encrypted data directly.

**Table 1.** Comparison of access times (seconds) for BAM and VCF file with Java and C libraries for plain and Crypt4GH-encrypted data (and index) files. Results are averaged over 11 runs

	C/htslib		Java/htsjdk	
	Plain	Crypt4GH	Plain	Crypt4GH
Full read (BAM)	14.35	17.48	23.20	30.46
Random queries (BAM)	98.81	120.89	119.31	170.95
Full read (VCF)	267.38	268.48	111.10	114.17
Random queries (VCF)	5.72	5.80	21.71	21.30

## 4 Discussion

We have defined an encrypted file container format that can be added as a standard input and output format to many existing biological libraries and tools, as demonstrated with htslib and htsjdk. Crypt4GH makes it possible to efficiently keep data (and index) files in an encrypted state that is directly accessible by current tools. With Crypt4GH, exchanging sensitive genetic data only requires the creation of a new header, whereas the data portion remains unchanged. This format also works with remote/API access, including GA4GH htsget (Kelleher *et al.*, 2019), enabling integration into end to end discovery, authorisztion and data access workflows.

## Supplementary Material

btab087_Supplementary_Data
