# Supplementary material for: Crypt4GH: a file format standard enabling native access to encrypted data
Source: Bioinformatics. 2021 Feb 5;37(17):2753–4. doi: 10.1093/bioinformatics/btab087 (PMC8522443; doi:10.1093/bioinformatics/btab087)
Supplement: btab087_Supplementary_Data [file btab087_supplementary_data.zip › Cleaned-Crypt4GH-v1-Product-Approval Security Questionaire.pdf]

Global Alliance
for Genomics & Health

Product-Approval Security Questionnaire

Work Stream Name: Large Scale Genomics

Product Name: Crypt4GH

Targeted Date for Steering Committee Review: September 03, 2019

Principal Point of Contact: Alexander Senf (ajsenf@gmail.com), Thomas Keane (tk2@ebi.ac.uk)

Security Point of Contact: Alexander Senf (ajsenf@gmail.com), Robert Davies (rmd@sanger.ac.uk), Frédéric Haziza (frederic.haziza@crg.eu)

1 Product Profile 1

2 Security Risk Assessment 2

3 Information Protection Objectives 8

4 Countermeasures Incorporated into Product Specification 8

5 Residual Risks 9

6 Vulnerability Reporting 9

1 Product Profile

Use Case Addressed: Sequencing raw data is typically stored and transferred in SAM/BAM/CRAM (reads) and VCF/BCF (genetic variants), specifications maintained by the Large Scale Genomics workstream of GA4GH. These files have associated indexes that enable rapid lookup of data from regions of the genome. These files can be encrypted using standard techniques/formats, however the data is typically decrypted on disk (or prior to transmission) to enable genomics tools to use it. We are proposing an encryption container format and implementations in two of the most widely used genomics libraries (htslib, htsjdk) that will enable data to remain encrypted at rest and during transport, and support existing genomic indexes without modification. We specifically address the situation where data is stored in a researcher's compute environment, awaiting, or post-analysis.

Product Type (standard, application, etc.): Standard

Approach: Authenticated encryption of data at rest and in transport, to prevent unauthorised access in potentially insecure or shared environments. Enable libraries and tools to directly access encrypted data, performing in-memory decryption of just the data being used at any given time to prevent unencrypted data storage on permanent storage.

Audience: Anyone who needs to store or use genomic data in a secure way.

2 Security Risk Assessment

What data security risks (confidentiality, integrity, and availability) were identified in the initial security review?

- **Confidentiality**: The data should only be readable by the intended user or users. Access is limited to intended users based on their private key. Crypt4GH creates an envelope around encrypted data. Each envelope is encrypted with user specific keys, allowing access to the data for that user. Access to the data encrypted in the Crypt4GH format requires access to the target user's private encryption key. Alternatively, the envelope can also be decrypted with a combination of the target user's public key, and the source's private encryption key. Confidentiality is dependent on the management of private keys. Key management is strictly speaking outside the scope of the Crypt4GH specs, but recommendations and reference implementations are in preparation.
- **Integrity (authenticity)**: It should not be possible for unauthorised parties to modify the file without the recipient noticing when they try to decrypt it. As a side benefit this also allows the detection of accidental modifications due to network transmission errors and file-system bit rot. The data portion is independently encrypted in 64K blocks to allow authenticated random access. Each block is authenticated by a MAC (Message Authentication Code) so any attempt to modify data inside a block will be detected. The MAC value protects both a block's data integrity as well as its authenticity. The format can currently make no guarantee about the integrity of the entire file. For the integrity/authenticity of a whole file to be supported it is necessary to guarantee the order and completeness of the blocks of the file. A future update of the specifications may add mechanisms by which this could be achieved (a possible solution would be to supply a block sequence counter as Associated Data when generating the MAC (AEAD, Authenticated Encryption with Associated Data) and by supplying an EOF segment with its own block number. Alternatively, the whole file could be signed by a third party tool such as PGP).
- **Availability**: This is not one of the security targets of the spec. The aim is to prevent unauthorized access of data, not to "guarantee timely access". (Reference implementations will be provided as part of the GA4GH approval process; but the product put forward for approval is the spec itself, not the accompanying software.)

Additional security risks:

- Key management issues
- Encryption Algorithm issues

<u>What additional risks were identified in the course of developing this product</u>?

System assumptions

- **Platform**: No specific assumption is made regarding the setting of where files are accessed; the purpose of Crypt4GH is to enable data to exist (at minimum at rest) on any setting, including a user's laptop, an institute's shared HPC/LSF cluster, Cloud VMs,

as well as more secure execution environments. The actual setting will affect possible attack vectors primarily based on who has access to the execution environment, and who may be able to observe main memory on these resources. “Using” a file implies a program reading the encrypted file from its source location, obtaining the private key of the target user, and performing on-demand decryption into main memory of byte ranges requested by the program during execution. Details regarding obtaining the target user private key are outside of the scope of the file format specifications, but are important to consider when using the format, because these details form one of the attack vectors for this format.

Programs that need to decrypt the format are likely to be run on the following platforms:

- A private batch queuing system.
  - This would be a collection of (usually many) machines that run jobs on behalf of users coordinated by a batch queuing system (e.g. lsf, slurm).
  - The machines will have access to shared file systems.
  - Many users will have access to the system.
  - The machines and file systems are owned and run by a single organisation to which the users running the jobs belong.
- A virtualized host ("cloud" machine). This could be either private or in a public for-hire service.
  - A management service allows hosts to be started rapidly. When the host has finished running its job, it is usually turned down immediately so the underlying hardware can be used for another task.
  - Hosts are usually virtual machines so more than one may be running at once on the underlying hardware, although it is possible to either request exclusive access or run “bare-metal” directly on the hardware itself.
  - Shared storage is frequently via block stores like CEPH, S3 or Google cloud storage rather than traditional POSIX filesystems.
  - In private clouds the host machines are owned and managed by the same organisation that users running jobs belong to.
  - In public for-hire services, users rent time on machines owned by another company (Amazon, Google, Microsoft, and many more). These companies also offer block storage solutions that are accessible over the web.
- A personal machine (e.g. a laptop)

The encrypted data may be stored on:

- A network-attached filesystem (NFS, CIFS, Lustre, etc.)
- A private "cloud" (CEPH, etc.)
- A public "cloud" file store (Amazon S3, Google cloud storage, etc.)
- Behind APIs (GA4GH DOS, GA4GH htsget server, etc.)
- An internal filesystem (local hard drive / SSD)
- A removable filesystem (external drive)

The encrypted data may also be put on file servers for transfer over the network.

Components

- Client: Program reading/writing the format
- Private key: Users' key needed to access the data
- Agent: Agent that grants access to the private key
- Keyring: Encrypted file containing the users' private key(s).

Identified Threats

- Passphrase guessing
    - Attack vector: An attacker who has both an encrypted file and a copy of the users' keyring can try to guess the keyring passphrase using a dictionary attack or other brute-force methods. Once the attacker has the passphrase, all files secured using keys in the keyring can be read.
    - Security weakness: Weak passphrase; the ability to rapidly test passphrase guesses.
    - Vulnerable component: Keyring encryption
    - Counter measures: Implementations should try to ensure the passphrase is strong. Use a slow key derivation function (e.g. scrypt or Argon2) to limit the rate of brute force attacks.
- Agent hijacking
    - Attack Vector: An attacker with sufficient privilege could connect to the agent process and use it to obtain the required decryption keys.
    - Security weakness: Ability of the agent to ensure only authorised users and processes are connecting to it.
    - Vulnerable component: Agent
    - Counter measures: It may be possible to use OS-specific features to limit which processes can connect to the agent (e.g. Linux namespaces).
      (This attack vector is outside the scope to the file format specifications, but it is important to consider to keep data secure in actual usage settings.)
- Impersonation of authorised user
    - Attack Vector: If an attacker can launch a process as a legitimate user, they may as a result be able to read an encrypted file.
    - Security weakness: Authenticating legitimate processes.
    - Vulnerable component: Agent.
    - Counter measures: Keep OS patch level updated to prevent elevation of privilege based attacks. Use a strong passphrase to access the system.
- Obtaining keys from running processes
    - Attack Vector: It may be possible to extract keys by examining the memory of running processes. There is also a risk of keys being leaked if the memory they are stored in is swapped out.
    - Security Weakness: Poorly-secured applications.

- Vulnerable component: Client and/or agent process.
- Counter measures: Keep keys in unswappable memory; securely zero the memory when no longer needed. Use operating-system features to disable debugging of processes with access to keys.

(This attack vector is outside the scope to the file format specifications, but it is important to consider to keep data secure in actual usage settings.)

- Broken encryption
  - Attack Vector: The encrypted data may remain sensitive for a very long time (if we consider the limit to be the lifetime of the patient involved). It is possible that the encryption could be broken over this period. This is especially true for most current public-key algorithms, which could be vulnerable to attacks using quantum computers.
  - Security weakness: Encryption found to be weak.
  - Vulnerable component: Encryption.
  - Counter measures: Update the specs/implementations if threats to the underlying encryption algorithm are detected. However, this still requires re-encrypting the vulnerable file, otherwise it would remain vulnerable.

Additionally, based on identified use case scenarios:

- Scenario part 1
  - Customer contacts scientific service representative (SSR) requesting sequencing of human samples in a new study (the sequencing service in this setup also includes long term access to the data product).
  - SSR requests user [Curve25519] public keys (people or automated systems) who require access to the sequencing data product
  - On receipt of the keys, potentially with key fingerprint checking (depending on local security policies), they are associated with the study (and/or samples) for which sequencing is to be performed. [Outside of the specifications; probably need to make recommendations].
  - On sequencing being completed, analysis is performed automatically which includes encrypting data products (cram, crai files) using the customer provided public keys for the appropriate study (and those of the analysis provider), as they are created.
  - Encrypted data products are placed in an archive from which the customer can retrieve them.

  <u>Risk(s):</u> Authentication of the target user public key; correspondingly, handling of the user private key.

  <u>Counter Measures:</u>User private key handling is outside the scope of this specification; it is up to the user to ensure secure key management. User public key is embedded with the submitted file; it could be checked against the public key exchanged via a separate

secure channel, if authentication of the user origin is desired. This is an operational question and falls outside the scope of this specification.

- Scenario part 2a
  - Customer's bioinformatician downloads a subset of data to their local machine/laptop to run qc metric generating tools on it and test analyses. The bioinformatician ensures a key server is running, the tools configured to use it and their private key (corresponding to the public one they sent to the sequencing provider) loaded into it.

  Risk: Handling of user private key.

  Counter Measures:User private key handling is outside the scope of this specification; it is up to the user to ensure secure key management.

- Scenario part 2b
  - Customer commissions 3rd party to provide bulk analysis
  - Analysis provider's public key (for this study) is provided to the sequencing provider who update the data products by adding the new key
  - Analysis provider distributes analysis over many compute nodes where key is distributed to and purged from automatically
  - Resulting [variant] analysis results e.g. gVCF are also encrypted on the fly with the customers public key and uploaded to the customer.
  - On analysis commission being completed the sequencing provider updates the original data products to remove the analysis providers key

  Risk: Authorisation -- who is allowed to access data?

  Counter Measures: This is decided by data owners, data access committees, etc. If the target user is an individual, their private key should not be shared. If the target is a lab/organisation, then key management will be crucial. [Key management is an implementation detail, and it is outside the scope of the file format spec.]

- Scenario part 3
  - Certain genomics regions are determined to be of interest to collaborators who have approval to access those genomic regions but no others.
  - sequencing provider updates the original data products, and customer updates variant analysis result files, to add the collaborators key in a manner which only allows access to those parts of the genome access is agreed for.
  - Collaborator is able to download those regions of both original and variant analysis with standard tools (cram/bam/vcf indexed), or download the whole file, but can only access data for the regions agreed.

Risk: Data leaks within single files.

Counter Measures: We decided not to allow for this use case. [Reason: to provide authenticated data in random/access & streaming scenarios we encrypt & MAC data in blocks of 64K. To retain efficient random access algorithms we don't allow for varying block sizes. The issue would therefore be all cases where a user is supposed to have access to some of the data within a block, but not another part. A secondary, pragmatic reason is that we don't want to force users to transfer large amounts of data to which they do not have been granted access. The solution (utilizing the random access capabilities of this format) is to create subsets of files in cases where access needs to be limited to subsets of data, using standard Crypt4GH enabled tools.]

Other risks identified:

- Encryption Envelope/Header: Public key encryption of the header is accomplished via Curve25519. A shared private key is generated via multiplication on the Curve25519 Elliptic Curve, according to (https://tools.ietf.org/html/rfc7748), but this procedure can produce identical results for more than one private/public key pair.

  Counter Measures: The solution is to introduce hashing to ensure uniqueness of the shared private key (according to https://libsodium.gitbook.io/doc/advanced/scalar_multiplication)

- Encryption blocks: data is encrypted in blocks of 64K using ChaCha20 (IETF specification) and authenticated using Poly1305 MACs. Each block is preceded by a 12 byte nonce, and the 16 byte MAC is appended to the data block. The encryption key is the same across the entire data. The risk is that a nonce value is reused within the same Crypt4GH file, leaking information about the encrypted data.

  Counter Measures: The solution is implementation-specific; close review of implementations of the standard need to be put in place to ensure that no nonce value is re-used.

- Data is encrypted and authenticated in blocks, specifically to enable streaming / random access / slicing use cases, where only a small part of the file is decrypted. Handling encryption and authentication in 64K blocks allows fine-grained access to files, being able to authenticate the data. Because each block is essentially encrypted independently, it is possible for an attacker to delete or re-order encrypted blocks of data. Data can't be read or modified, but new blocks can be added if the attacker knows the recipient's public key (and the source's private key). But the file can be rendered unusable without this being able to be detected by the block-wise authentication scheme, until the file is decrypted.

Counter Measures: The encryption algorithm (ChaCha20-IETF-Poly1305) is an Authenticated Encryption with Associated Data (AEAD) algorithm, which allows for Associate Data to be supplied when generating the MAC. This enables a file to be encrypted by supplying a block counter value, so that blocks can only be decrypted when their position in the sequence is known. This prevents unknowingly re-ordered blocks from being decrypted, because they fail the MAC check. For file completeness an EOF block with its own counter value can enable checks to ensure that no blocks were dropped or added. If sequence and completeness of blocks can be validated, then the integrity and authenticity of the whole file can be verified.

- Multiple users of the same data: one of the features of the proposed standard is to allow sharing of encrypted data to individual users, without having to re-encrypt all the data content multiple times with different keys. Instead, user specific headers are generated. If two users (Alice, Bob) have been given access to the same data, both will share the means to learn the symmetric data encryption key. That might allow user Bob to modify the data content of user Alice's file, without Alice being able to detect that the file was tampered with.

  Counter Measures: This is outside of the scope of what we want to achieve with this format, which is primarily to prevent unauthorized data access. For this purpose we assume users being granted access to file to be trustworthy. We are thinking about this as a feature in a future version.

3 Information Protection Objectives

Which of the following information protection objectives were assigned to each security risk?

1) **Eliminate vulnerability**
2) **Restrict access to target**
3) **Reduce ease of exploitation**
4) **Reduce pay-off value of exploitation**
5) **Detect attempts to exploit**
6) **Take action when exploitation attempt is detected**

This section is covered in the Attackers Model document:
https://docs.google.com/document/d/1kMpw9fgq204q8veb3o7qDPKqaRLtWLsb2_6TKDdAYiU/edit#heading=h.c0b9vv9eixji

4 Countermeasures Incorporated into Product Specification

Identify the countermeasures incorporated, and describe how each countermeasure contributes to meeting the protection objectives.

The product specification is for an encrypted file format. Every effort has been made to use best and proven available algorithms (Curve25519, ChaCha20, Poly1305) and construct the format not to expose any weaknesses.
The approach is to periodically review all of the standards used in the specification, to identify any vulnerabilities.

A secondary aspect is to ensure that implementations of the specifications are secure, and don't allow for any weak spots.

5 Residual Risks

Identify any vulnerabilities that remain, describe how they might be exploited, and explain why the residual risk is deemed acceptable.

If the specifications are implemented properly the remaining risk is related to handling of private keys. Recommendations are in place for handling the keys, but this is, strictly speaking, outside of the scope of the specifications, but part of any system built to handle secure data. One remaining risk is the behaviour of authorized user, who may use their knowledge of the encryption parameters to tamper with the encrypted data. This is also outside of the scope of the specifications.

Reference implementations are built upon proven open source libraries.

6 Vulnerability Reporting

What mechanisms have you implemented to enable users of this product to report discovered vulnerabilities? What is the plan for responding to vulnerability reports?

This product is a file format specification. Vulnerabilities can occur if weaknesses in the algorithms chosen for this format are discovered (e.g. ChaCha20-Poly1305, Curve25519 Key Exchange with bcrypt hashing).

If any vulnerabilities in any of the algorithms specified in the specification are discovered or reported, the spec maintainers will:

- Evaluate the scope of the vulnerability (e.g. does it affect any file encrypted with the algorithm? etc.)
- Analyze the severity - does it pose a risk to any currently encrypted data? Is there a known exploit already available? Does it allow unauthorized access to the whole file or just certain blocks within the file? Etc.
- Applicability: Is it dependent on the size of the encrypted data? Does it require access to the local machine during use? Is it a vulnerability of a library implementation, or of an algorithm? etc.

Based on the outcome of the analysis different actions are required:

- If the vulnerability is related to any of the algorithms specified in the specs, and is severe, then the spec itself must be updated, replacing any vulnerable algorithms with

new algorithms. A new major spec version must be created, approved by GA4GH, and any previous spec will be deprecated. This would be a change that breaks compatibility. Any known user and implementer of the spec will be contacted.

- If the vulnerability is related to any of the algorithms specified in the specs, but it is not severe, then the spec itself will be updated, replacing any vulnerable algorithms with new algorithms. This change would be implemented more slowly to allow any users to prepare for a new version of the algorithm, allowing them to continue use of the current version if they are comfortable that the vulnerability does not pose a security threat to their use case. Any known user and implementer of the spec will be contacted in any case.
- If the vulnerability is related to any library or implementation of an algorithm, but not to the algorithm itself, then the implementation guidelines will be updated to ensure that only secure libraries are used in implementations of the specification. Any implementers known to use affected libraries will be contacted. If any of the reference implementations are affected, they will be updated and previous versions will be deprecated. General notices will be posted in the respective code repositories, so that any users of the reference implementations are notified. The implementation guide will specify the recommended course of action based on the severity of the vulnerability - this can range from advice/suggestions to mandatory replacement of affected libraries.

Reporting of any identified vulnerabilities should happen to the security points of contact identified in this document, as well as to the general GA4GH Security point of contact: security-notification@ga4gh.org.

[Any vulnerabilities in implementations of this standard should be reported to the implementers; in case of the reference implementation this is the same point of contact as for the specification. In case of 3rd party implementations, it would be good for GA4GH to at least know about this; so reporting it to the general GA4GH Security point of contact (security-notification@ga4gh.org) is encouraged.]
