# Supplementary material for: Crypt4GH: a file format standard enabling native access to encrypted data
Source: Bioinformatics. 2021 Feb 5;37(17):2753–4. doi: 10.1093/bioinformatics/btab087 (PMC8522443; doi:10.1093/bioinformatics/btab087)
Supplement: btab087_Supplementary_Data [file btab087_supplementary_data.zip › Crypt4GH Supplementary Data (3).pdf]

Implementations and Benchmarking

In this supplement we describe the implementations of the Crypt4GH format that were developed, and provide initial benchmarking results.

URLs and implementations specified in this supplementary data page is the code that was developed during the development of the spec, and may change.

0 Crypt4GH Standard

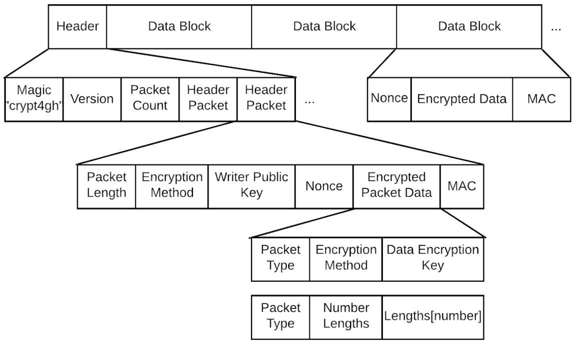

Supp. Fig. 1. Crypt4GH File Structure. A user-specific header and the data portion, encrypted symmetrically in 64K segments. Header and data portion are encrypted using the same encryption algorithm, but different keys. The header key is derived via X25519 ECDH key exchange with a user's public key. The data key is randomly generated and embedded in the header. **Crypt4GH Data Block:** A 12 byte nonce, followed by 64KB of encrypted Data, followed by a 16-byte MAC over the encrypted data. **Crypt4GH Header:** Three plain text fields to identify the format and version, plus one or more encrypted header packets, and a count of the number of header packets. There are several types of header packets. **Crypt4GH Header Packet:** Header Packets are encrypted for a user. Therefore, in addition to the packet length, the encryption method used, and the writer's public key must both be specified. The payload of the packet is then encrypted and contains either the information to access the data portion (encryption method used, and encryption key) or the edit list.

A Crypt4GH file consists of a header and a data portion (Supp. Fig. 1). The data portion is the original content that is to be encrypted. The header is encrypted with an individual recipient's public key and contains the secret symmetric encryption key necessary to access the data portion. Both a private and a public key from two different sources are required to encrypt and decrypt the header. The private key used for header encryption (in conjunction with a user's public key) is usually randomly generated, and the derived public key component is stored in the header packet. The recipient decrypts header packets using their own private key, and the encrypter's public key from the header.

A message authentication code (MAC) can be used as an integrity check. It is based on a shared secret key and prevents decryption if the recipient cannot validate it. Using authenticated encryption in individual segments mirrors solutions like Transport Layer Security (TLS)

(Rescorla, 2018) and prevents undetected modification of segments. Dividing the file into fixed-size (except at the end) blocks enables random-access into the encrypted file. The format is also designed to support streaming reads and writes.

The header contains unencrypted fields identifying the format with magic and version numbers, as well as a count of header packets. The header packets are encrypted with the help of a user's public key and contain information necessary to access the data portion of the file. There are two types of header packets. One contains the encryption key for the data portion of the file, and the other identifies subsets of data within the 64KB data blocks (edit list).

One of the goals of Crypt4GH is to delay decryption of data to the point where the data is being analysed. In that context it sometimes becomes necessary to create subsets (e.g. a genomic locus) of the data that do not align with the 64KB boundaries of the encrypted data blocks. Creating a valid BAM or CRAM file may require some bytes in a data block to be ignored after decryption. The edit list allows for this to be specified in the header.

Multiple encryption methods can be specified, whilst only one is currently supported: ChaCha20-Poly1305 (Bernstein, 2008). This simplifies implementations while allowing the current choice to be replaced in a future revision if it is found to have any vulnerabilities. The criteria for choosing encryption methods included having strong security guarantees, widespread library support, and being used in other common standards such as TLS version 1.3.

1 Implementations

We provide two types of implementation: On the one hand there are stand-alone tools that allow users to encrypt or decrypt data in the Crypt4GH format. On the other hand there are plugins or extensions of genomic libraries, that enable users to read and write Crypt4GH data directly from an application, without having to use a separate program decrypting data first.

1.1 Stand Alone Tools

These tools are used to encrypt or decrypt data only.

1.1.1 Java

Java 13 implementation is available at: https://github.com/uio-bmi/crypt4gh

A Java 11 fork is available at: https://github.com/AlexanderSenf/crypt4gh-1

1.1.2 Python

A Python 3.6+ implementation is available at: https://github.com/EGA-archive/crypt4gh

1.1.3 Library Implementations

These add Crypt4GH as a standard input/output format to genomic tools. The goal for this format is to enable native usage of encrypted data so we expect this to be the more important use case of the two.

We are currently actively working on this code to improve its performance.

1.1.4 htslib

This implementation is available here: https://github.com/samtools/htslib-crypt4gh

The htslib implementation is written in C and is available as a plugin for samtools. The instructions to build a samtools version that can natively read and write Cryt4GH data are included in this library.

1.1.5 htsjdk

This implementation is available here: https://github.com/AlexanderSenf/htsjdk

This proof-of-concept implementation is written in Java 11 and provides a Crypt4GH-enabled input (SeekableStream) class. Any standard input stream can wrap the Crypt4GH-enabled stream, which allows all htsjdk functionality to work natively with Crypt4GH data.

The proof-of-concept implementation is built using native Java 11 cipher algorithms (ChaCha20-Poly1305). We are aiming to merge Crypt4GH functionality with htsjdk (as well as Picard and GATK) to coincide with the move of these libraries to Java 11.

2 Initial Benchmarks

Benchmarking is done with the library implementations, to assess how much overhead would be added if a workflow would switch from plain input data to Crypt4GH encrypted data files. These tests only measure I/O performance, no work is performed on the data. In this sense these are worst-case measurements in terms of overhead.

BAM file benchmarks were performed on file `6929_4#44.bam` as input (1 GB; https://www.ebi.ac.uk/ena/browser/view/ERR065185). VCF file benchmarks were performed on file `ALL.chr13.phase3_shapeit2_mvncall_integrated_v5a.20130502.genotypes.vcf.gz` (0.55 GB; ftp://ftp.1000genomes.ebi.ac.uk/vol1/ftp/release/20130502/ALL.chr13.phase3_shapeit2_mvncall_integrated_v5a.20130502.genotypes.vcf.gz)

The benchmark is also described here: https://github.com/AlexanderSenf/htsjdk_crypt4gh_test

2.1 htslib Benchmarks

This test encrypts the source file first, and then performs a simple view operation on the entire content of the file, incurring decompression overhead, and in the case of Crypt4GH, also decryption overhead.

2.1.1 Results Table (htslib and htsjdk)

The tests performed are:

- Iterate through all records sequentially
- Perform 1000 random queries across the whole file, and accessing the results

The tests record the average over 11 runs. Query parameters were stored in BED like files, to ensure identical test runs across repeats and platforms.

	**C/htslib**		**Java/htsjdk**	
	**Plain (s)**	**Crypt4GH (s)**	**Plain (s)**	**Crypt4GH (s)**
BAM file: Iterate through all records	14.359	17.485	23.203	30.461
BAM file: 1000 random queries	98.816	120.898	119.319	170.955
VCF file: Iterate through all records	267.383	268.481	111.107	114.178
VCF file: 1000 random queries	5.729	5.802	21.719	21.301

Table 1: Comparison of access times for BAM and VCF file with Java and C libraries for plain end Crypt4GH-encrypted data (and index) files.

Observations:

- VCF times seem to be dominated by gzip decompression times
